# Early postoperative serum aspartate aminotransferase for prediction of post-hepatectomy liver failure

**DOI:** 10.1186/s13741-022-00283-y

**Published:** 2022-10-07

**Authors:** Watoo Vassanasiri, Narongsak Rungsakulkij, Wikran Suragul, Pongsatorn Tangtawee, Paramin Muangkaew, Somkit Mingphruedhi, Suraida Aeesoa

**Affiliations:** Department of Surgery, Faculty of Medicine, Ramathibodi Hospital, Mahidol University, 270 Praram VI Road, Ratchathewi, Bangkok, 10400 Thailand

## Abstract

**Background:**

Post-hepatectomy liver failure (PHLF) is a serious complication of hepatectomy. The current criteria for PHLF diagnosis (ISGLS consensus) require laboratory data on or after postoperative day (POD) 5, which may delay treatment for patients at risk. The present study aimed to determine the associations between early postoperative (POD1) serum aminotransferase levels and PHLF.

**Methods:**

The medical records of patients who underwent hepatectomy at Ramathibodi Hospital from January 2008 to December 2019 were retrospectively examined. Patients were classified into PHLF and non-PHLF groups. Preoperative characteristics, intraoperative findings, and early postoperative laboratory data (serum AST, ALT, bilirubin, and international normalized ratio (INR) on POD0 to POD5) were analyzed.

**Results:**

A total of 890 patients were included, of whom 31 (3.4%) had PHLF. Cut-off points for AST of 260 U/L and ALT of 270 U/L on POD1 were predictive of PHLF. In multivariate analysis, AST > 260 U/L on POD1, ICG-R15, major hepatectomy, blood loss, and INR were independently associated with PHLF.

**Conclusions:**

Early warning from elevated serum AST on POD1, before a definitive diagnosis of PHLF is made on POD5, can help alert physicians that a patient is at risk, meaning that active management and vigilant monitoring can be initiated as soon as possible.

## Background

Post-hepatectomy liver failure (PHLF) is the most serious complication and a common cause of mortality following hepatic resection (Russell, 2015; Bagante et al., 2019). The current mortality rate from hepatectomy is significantly lower than it has been in the past (Søreide & Deshpande, 2021). This is due to increased understanding of liver physiology, improved guideline for hepatectomy patient selection, and advances in surgical technique, surgical instruments, and critical care management (Qadan et al., 2016). However, PHLF still occurs and remains a major concern for hepatic surgeons because of the lack of specific treatment modalities (Qadan et al., 2016). Using the definition in the international consensus of the Liver Surgery Group (ISGLS consensus), the incidence of the PHLF is 1.2–32% (Søreide & Deshpande, 2021; Qadan et al., 2016; Rungsakulkij et al., 2019).

Predictors of PHLF can be categorized into three main groups with regards to the timing the predictor may be present: preoperative, intraoperative and postoperative predictors. Many studies have tried to identify preoperative risk factors for the development of PHLF (Qadan et al., 2016; Dasari et al., 2019; Honmyo et al., 2021). Low future liver remnant volume and the presence of preoperative portal hypertension are the most commonly identified significant preoperative predictors (Yoshino et al., 2021). However, poor preoperative risk scores did not always prevent surgeons from performing hepatectomy (Olthof et al., 2016).

Intraoperative factors such as prolonged operative time, liver ischemia, intraoperative blood loss, and extent of hepatectomy have been shown to affect the incidence of PHLF (Qadan et al., 2016; Roberts et al., 2013) and may inform the attending physicians that close monitoring is warranted in certain patients.

With regard to postoperative predictors, according to the ISGLS consensus (Rahbari et al., 2011), which defined PHLF as postoperative-acquired deterioration in the ability of the liver to maintain its synthetic, excretory, and detoxifying functions (such function failures are characterized by increased INR (or requirement of clotting factors to maintain normal INR) and hyperbilirubinemia (according to normal cut-off levels defined by the local laboratory) on or after postoperative day 5 (POD5)), the diagnosis of PHLF cannot be made until POD5 or after. This leaves a 5-day gap between the operation and the diagnosis of PHLF where if early (before POD5) postoperative predictors can provide accurate warnings that certain patients might be at risk of PHLF, then physicians may be able to more actively manage them (Roberts et al., 2013; Grat et al., 2013). Postoperative factors such as elevated serum transaminase and bilirubin level have also been shown to predict the incidence of PHLF (Qadan et al., 2016; Roberts et al., 2013). Elevated serum transaminase and bilirubin levels indicate hepatocellular injury and affected patients require further assessment and potential treatment (Kwo et al., 2017). The relationships between peak post-operative serum transaminase levels and overall morbidity and mortality following hepatectomy were explored in several studies (Olthof et al., 2016; Boleslawski et al., 2014; Bhogal et al., 2016). However, the studies yielded contradictory findings and their outcomes of interest were overall morbidity and mortality rather than PHLF in particular. Only one report from Yu et al. (Yu et al., 2018) described an association of prolonged serum transaminase elevation with severe PHLF, but the findings were restricted to a PHLF cohort and were not compared with a non-PHLF group. There is limited evidence on the associations between serum transaminase levels and PHLF. Thus, the aim of the present study was to determine the associations of postoperative serum transaminase level with PHLF and the usefulness of early postoperative liver function testing in clinical practice.

## Methods

The study design was a case-control study. A total of 920 consecutive patients who underwent hepatectomy at the Department of Surgery, Ramathibodi Hospital, Mahidol University, Bangkok, Thailand, from January 2008 to December 2019 were retrospective analyzed. Nine subjects were excluded due to duplicated or incorrect operative records in the system. Patients with no records of PHLF and liver test data on postoperative day (POD) 5 were excluded from the study. A further 21 patients were excluded (all due to incomplete data on postoperative liver test) resulting in the final sample size of 890 patients. The study protocol was approved by the Institutional Ethical Committee at Faculty of Medicine, Ramathibodi Hospital, Mahidol University, Thailand (protocol number, MURA2020/971). At Ramathibodi Hospital, candidates for surgery were routinely evaluated by a multidisciplinary team comprising surgeons, gastroenterologists, medical oncologists, radiologists, and interventionists. All patients underwent preoperative cross-sectional dynamic imaging using triple-phase computed tomography (CT) or magnetic resonance imaging (MRI). Routine blood examinations were performed, including complete blood count (CBC), coagulogram, liver function tests (LFT), kidney function tests, and preoperative serum alpha-fetoprotein level. Preoperative characteristics including the American Society of Anesthesiologists class, evidence of viral hepatitis B or C infection, and smoking status were recorded. A preoperative indocyanine green retention test at 15 min (ICG-R15) was also performed. Preoperative biliary intervention was defined as history of percutaneous or endoscopic biliary drainage or any previous operative procedures involving the biliary tract. In our center, the extent of liver resection was based on the liver functional reserve, determined mainly in accordance with the Makuuchi’s criteria (Miyagawa et al., 1995) and occasionally by volumetric CT analysis. The Makuuchi’s criteria included presence of preoperative ascites, Child–Pugh score, bilirubin level, and ICG-R15 value. The albumin-bilirubin (ALBI) score was collected. The formula for ALBI scoring system relies on the following equation: ALBI score = (log10 bilirubin [μmol/L] × 0.66) + (albumin [g/L] × − 0.0852). As a result, ALBI grades 1, 2, and 3 were developed as follows: ALBI score ≤ − 2.60 (ALBI grade 1), > − 2.60 to ≤ − 1.39 (ALBI grade 2), and > − 1.39 (ALBI grade 3) (Johnson et al., 2015).

### Perioperative and postoperative method

The perioperative patient care protocol in our center was described in a previous report (Rungsakulkij et al., 2019). The perioperative data collected for analysis includes diabetes mellitus, hypertension, dyslipidemia (DLP), hepatitis B virus (HBV), hepatitis C virus, smoking, operative time, and intraoperative blood loss. All patients were admitted to the intensive care unit after hepatectomy for postoperative monitoring and care by intensive care physicians. Liver segments were defined according to the Brisbane classification (Strasberg et al., 2000). Major hepatectomy was defined as the removal of four or more segments.

On POD0-3 and POD5, biochemical laboratory examinations were routinely performed, including CBC, LFTs, and coagulogram. After POD5, biochemical laboratory examinations were only performed if the attending physician deemed necessary. The postoperative biochemical laboratory data collected for analysis were serum creatinine, total bilirubin, alanine aminotransferase (ALT), aspartate aminotransferase (AST), alkaline phosphatase (ALP), serum albumin (Alb), INR, platelet, and serum phosphate (PO4) were collected.

### Postoperative complications

The occurrence of PHLF was determined in accordance with the criteria in the ISGLS consensus (Rahbari et al., 2011). Based on this criteria PHLF was defined as a postoperatively acquired deterioration in the ability of the liver to maintain its synthetic, excretory, and detoxifying functions, which are characterized by an increased INR and concomitant hyperbilirubinemia on or after postoperative day 5. Increased INR and hyperbilirubinemia are defined according to the normal range of cut-off levels of the local laboratory, which the cut-off levels at our institution are 1.2 mg/dL for total bilirubin and 1.17 for INR. This definition applies to patients with normal and abnormal preoperative liver function. If INR or serum bilirubin was abnormally elevated preoperatively, PHLF was defined as increasing INR (or decreasing prothrombin time) and increasing serum bilirubin on or after POD5 compared with the values of the previous day. Other obvious causes for the observed biochemical and clinical alterations such as biliary tract obstruction were ruled out. Postoperative mortality was recorded as 90-day mortality and in-hospital mortality.

### Follow-up

For malignant lesions, patients were followed up at an outpatient clinic every 3–6 months after surgery and routinely underwent imaging (ultrasonography, CT, MRI) and blood tests. Recurrent disease was defined as the presence of new tumors found on imaging (CT or MRI) during the follow-up period. For benign lesions, patients were followed up at an outpatient clinic at intervals deemed appropriate by the attending physician.

### Statistical analyses

For the patient characteristics, continuous variables were analyzed using the Student’s *t* test, and categorical variables were analyzed using the chi-square or Fisher’s exact test. A *p* value of < 0.05 was considered statistically significant. The potential risk factors were analyzed by univariate and multivariate analyses using a Binary logistic regression model with stepwise and best subset approach for variable selection (Zhang, 2016). Odds ratios (OR) and 95% confidence intervals (CI) were computed to assess the strength of the associations between the various factors and PHLF. A *p* value of < 0.05 was considered statistically significant. Analyses were performed using STATA program version 14 (StataCorp, College Station, TX, USA). The cut-off value of AST, ALT, and INR post-hepatectomy were determined by receiver operating characteristic (ROC) curve analysis. The values that provide the most accuracy for PHLF prediction were selected as cut-off values. Post-hoc power analysis for two-sample comparison of proportions to detect a statistical alpha of 5% significant level (two-sided) revealed a power of 0.83.

## Results

### Patient characteristics and perioperative status

Of the 890 patients who underwent hepatectomy from January 2008 to December 2019, 31 patients (3.4%) had PHLF. Of the 31 patients with PHLF, 17 (54.8%), 9 (29.0%), and 5 (16.3%) patients had PHLF grade A, B and C respectively. The clinicopathological characteristics of the cohort are summarized in Table 1. The PHLF group contained more men than women (67.7 vs. 32.2%, *p* = 0.032) and more smokers than nonsmokers (55.1 vs. 31.2%, *p* = 0.007). The PHLF group had significantly worse ALBI score compared to the non-PHLF group (percentage of ALBI grade I, II, and III were 3.2, 45.2, and 51.6% in the PHLF group and 1.3, 71.6, and 27.2% in the non-PHLF group respectively). The PHLF group had significantly higher levels of certain preoperative laboratory data than the non-PHLF group, namely serum AST (71 vs 41 U/L, *p* = 0.001), ALT (66 vs 45 U/L, *p* = 0.002), and ALP (248 vs 112 U/L, *p* = 0.002). The PHLF group had higher incidence rate of cholangiocarcinoma than non-PHLF group (32.3 vs 5.8%, *p* < 0.001). The ICG R15 value was higher in the PHLF group compared with the non-PHLF group (18.5 vs 13.9%, *p* = 0.025). Most patients in the PHLF group received hepatic lobectomy, while most patients in the non-PHLF group underwent minor hepatectomy (80.6% lobectomy and 12.9% minor hepatectomy in the PHLF group vs 24.9% and 63.6%, respectively, in the non-PHLF group). The operative time was longer in the PHLF group compared with the non-PHLF group (9.29 vs 5.83  h, *p* < 0.001). The PHLF group also experienced greater intraoperative blood loss and longer clamp time than the non-PHLF group (blood loss 1600 vs. 600 ml, *p* < 0.001, mean clamp time 72 vs. 57 min, *p* = 0.013).

**Table 1:** Patient’s characteristic

Variable	Total (***n*** = 890)	Non-PHLF (***n*** = 859)	PHLF (***n*** = 31)	***p*** value
Age (year), mean + SD	56.19 + 13.88	56.19 + 13.99	56.19 + 10.55	0.998
Gender, *n* (%)				
Male	435(48.88)	414(48.20)	21(67.74)	0.032
Female	455(51.12)	445(51.80)	10(32.26)	
Comorbidity, *n* (%)				
DM	192(21.57)	186(21.65)	6(19.35)	0.760
HT	374(42.02)	366(42.61)	8(25.81)	0.063
DLP	202(22.70)	200(23.28)	2(6.45)	0.028
HBV	209(23.48)	201(23.40)	8(25.81)	0.756
HCV	74(8.31)	72(8.38)	2(6.45)	0.702
Smoking, *n* (%) *n* = 881				
No	599(67.99)	586(68.78)	13(44.83)	0.007
Yes	282(32.01)	266(31.22)	16(55.17)	
Platelet count × 10^3^, mean + SD, *n* = 830	235 + 88	234 + 89	240 + 87	0.712
Creatinine (mg/dL), median (IQR) *n* = 796	0.83(0.67, 1.01)	0.83(0.68, 1.00)	0.84(0.74, 1.02)	0.834
ALBI score, *n*(%) *n* = 819				
ALBI grade I (≤− 2.60)	11(1.34)	10(1.27)	1(3.23)	0.006
ALBI grade II (>− 2.60 to ≤− 1.39)	578(70.57)	564(71.57)	14(45.16)	
ALBI grade 3 (>− 1.39)	230(28.08)	214(27.16)	16(51.61)	
Preoperative liver function data				
TB (mg/dL), mean ± SD, *n* = 826	0.99 + 1.86	0.99 + 1.89	1.06 + 0.93	0.698
ALT(U/L), mean ± SD *n* = 808	46 + 37	45 + 37	66 + 42	0.002
AST(U/L), mean ± SD, *n* = 828	42 + 35	41 + 34	71 + 46	0.001
ALP(U/L), mean ± SD *n* = 826	117 + 88	112 + 74	248 + 223	0.002
Alb(g/L), mean ± SD, *n* = 867	37.06 + 5.05	37.13 + 5.06	35.34 + 4.64	0.053
INR, mean ± SD, *n* = 798	1.04 + 0.11	1.03 + 0.11	1.09 + 0.15	0.059
Preoperative neoadjuvant, *n* (%)				
No	748(84.04)	724(84.28)	24(77.42)	0.305
Yes	142(15.96)	135(15.72)	7(22.58)	
Diagnosis, *n* (%)				
Hepatocellular carcinoma	148(16.63)	143(16.65)	5(16.13)	0.000
Cholangiocarcinoma	60(6.74)	50(5.82)	10(32.26)	
Colorectal liver metastases	209(23.48)	203(23.63)	6(19.35)	
Other malignancy	80(8.99)	79(9.20)	1(3.23)	
Donor hepatectomy	91(10.22)	90(10.48)	1(3.23)	
Benign tumor	302(33.93)	294(34.23)	8(25.81)	
ICG R15 (%), mean ± SD *n* = 537	14.19 + 10.18	13.98 + 9.95	18.55 + 13.38	0.025
Type operation, *n* (%)				
Lobectomy	240(26.97)	215(25.03)	25(80.65)	0.000
Sectionectomy	100(11.24)	98(11.41)	2(6.45)	
Segmentectomy	75(8.43)	75(8.73)	0	
Limited resection	475(53.37)	471(54.83)	4(12.90)	
Type operation, *n* (%)				
Minor	650(73.03)	644(74.97)	6(19.35)	0.000
Major	240(26.97)	215(25.03)	25(80.65)	
Operative time (h), mean ± SD *n* = 877	5.95 + 2.36	5.83 + 2.23	9.29 + 3.24	0.000
Blood loss (ml), median (IQR) *n* = 877	600(300, 1000)	600(300, 1000)	1600(1000, 3000)	0.000
Clamp time (min), mean ± SD *n* = 620	58.46 + 32.73	57.76 + 32.66	72.48 + 32.29	0.013
PHLF grading, *n*(%) *n* = 31				
Non-PHLF	–	859(100)	–	–
Grade A	–	–	17(54.8)	–
Grade B	–	–	9(29.03)	
Grade C	–	–	5(16.13)	
Recurrence, *n* (%)				
No	681(76.52)	663(77.18)	18(58.06)	0.014
Yes	209(23.48)	196(22.82)	13(41.94)	
Death within 90 days, *n* (%)				
No	884(99.33)	854(99.42)	30(96.77)	0.192
Yes	6(0.67)	5(0.58)	1(3.23)	

*PHLF* post-hepatectomy liver failure, *DM* diabetes mellitus, *HT* hypertension, *DLP* dyslipidemia, *HBV* hepatitis B virus, *HCV* hepatitis C virus, *ALBI* albumin-bilirubin, *ICG R15* indocyanine green retention test at 15 min, *TB* total bilirubin, *ALT* alanine aminotransferase, *AST* aspartate aminotransferase, *Alb* albumin, *INR* international normalized ratio

### Analysis of postoperative serum transaminase levels and their cut-off points

Comparisons of postoperative biochemical data by day between the PHLF group and the non-PHLF group are shown in Table 2. The PHLF group had significantly higher serum AST on POD0 (1063 vs 279 U/L, *p* = 0.011), POD1 (1567 vs 326 U/L, *p* = 0.001), POD2 (1075 vs 271 U/L, *p* = 0.009) and POD3 (473 vs 120 U/L, *p* = 0.001). The PHLF group also had significantly higher serum ALT on POD0 (748 vs 234 U/L, *p* = 0.007), POD1 (971 vs 295 U/L, *p* = 0.001), POD2 (823 vs 307 U/L, *p* = 0.008), and POD3 (561 vs 193 U/L, *p* = 0.002). Consistently, the PHLF group had significantly higher serum TB on POD0 (2.78 vs 1.66 mg/dL, *p* = 0.007), POD1 (3.83 vs 1.84 mg/dL, *p* < 0.001), POD2 (4.25 vs 2.52 mg/dL, *p* = 0.001), and POD3 (5.07 vs 1.85 mg/dL, *p* < 0.001) as well as significantly higher INR on POD0 (1.28 vs 1.14, *p* < 0.001), POD1 (1.41 vs 1.19, *p* < 0.001), POD2 (1.47 vs 1.25, *p* < 0.001), and POD3 (1.46 vs 1.18, *p* < 0.001).

**Table 2:** Postoperative biochemical data

Variable	Total (***n*** = 890)	Non-PHLF failure (***n*** = 859)	PHLF failure (***n*** = 31)	***p*** value
AST (U/L), mean ± SD				
Day 0, *n* = 766	306 + 424	279 + 297	1063 + 1481	0.011
Day 1, *n* = 796	373 + 609	326 + 435	1567 + 1907	0.001
Day 2, *n* = 412	322 + 550	271 + 379	1075 + 1463	0.009
Day 3, *n* = 732	135 + 210	121 + 170	473 + 535	0.001
POD1 AST(U/L), *n* (%) *n* = 796				
AST ≤ 260	469(58.92)	466(60.84)	3(10.00)	0.000
AST > 260	327(41.08)	300(39.16)	27(90.00)	
ALT (U/L), mean ± SD				
Day 0, *n* = 749	252 + 359	234 + 308	748 + 906	0.007
Day 1, *n* = 784	322 + 431	295 + 368	971 + 995	0.001
Day 2, *n* = 401	342 + 467	307 + 395	823 + 926	0.008
Day 3, *n* = 728	208 + 252	193 + 214	561 + 601	0.002
POD1 ALT (U/L), *n*(%) *n* = 784				
ALT ≤ 270	501(63.90)	494(65.60)	7(22.58)	0.000
ALT > 270	283(36.10)	259(34.40)	24(77.42)	
TB (mg/dL), mean ± SD				
Day 0, *n* = 766	1.70 + 3.03	1.66 + 3.06	2.78 + 1.98	0.007
Day 1, *n* = 797	1.92 + 3.57	1.84 + 3.58	3.83 + 2.56	0.000
Day 2, *n* = 413	2.63 + 6.53	2.52 + 6.72	4.25 + 1.87	0.001
Day 3, *n* = 748	1.98 + 6.49	1.85 + 6.57	5.07 + 2.56	0.000
INR, mean ± SD				
Day 0, *n* = 772	1.14 + 0.14	1.14 + 0.13	1.28 + 0.18	0.000
Day 1, *n* = 768	1.20 + 0.13	1.19 + 0.12	1.41 + 0.16	0.000
Day 2, *n* = 366	1.26 + 0.16	1.25 + 0.14	1.47 + 0.18	0.000
Day 3, *n* = 424	1.19 + 0.16	1.18 + 0.14	1.46 + 0.24	0.000
Alb (g/L), mean ± SD				
Day 0, *n* = 853	28.51 + 4.98	28.58 + 4.95	26.64 + 5.50	0.033
Day 1, *n* = 825	27.76 + 3.89	27.76 + 3.84	27.68 + 5.18	0.937
Day 2, *n* = 741	27.52 + 4.18	27.47 + 4.15	28.51 + 4.88	0.179
Day 3, *n* = 558	28.12 + 4.06	28.07 + 4.02	29.07 + 4.73	0.182
ALP (U/L), mean + SD				
Day 0, *n* = 766	86 + 52	85 + 50	128 + 92	0.022
Day 1, *n* = 797	81 + 46	78 + 42	132 + 89	0.003
Day 2, *n* = 412	82 + 51	78 + 38	137 + 126	0.023
Day 3, *n* = 733	86 + 50	84 + 44	140 + 111	0.009

*PHLF* post-hepatectomy liver failure, *TB* total bilirubin, *ALT* alanine aminotransferase, *AST* aspartate aminotransferase, *Alb* albumin, *INR* international normalized ratio

**Fig. 1:**
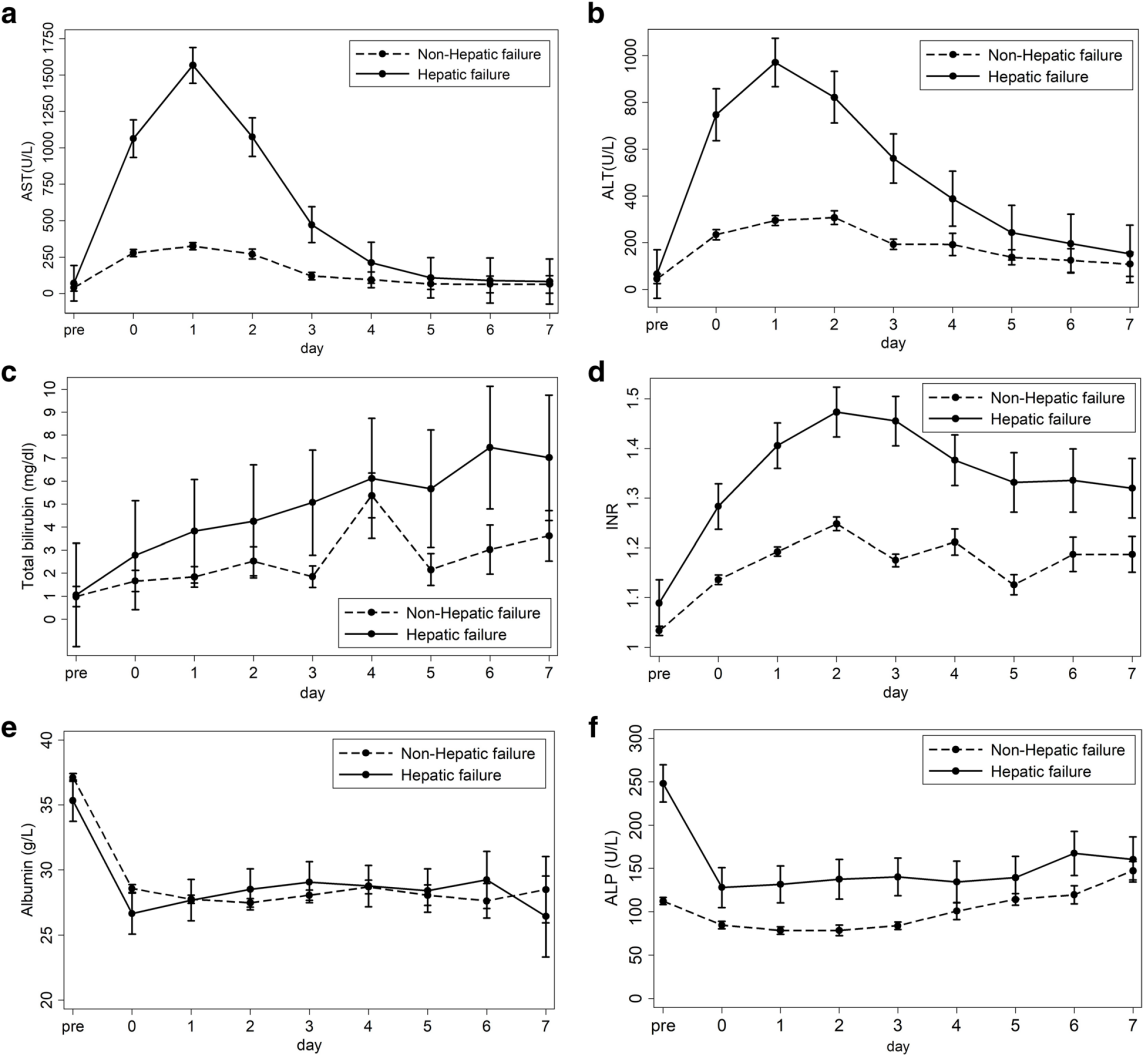
Kinetics of the postoperative serum transaminase levels. AST, aspartate aminotransferase; ALT, alanine aminotransferase. **a** AST, aspartate aminotransferase. **b** ALT, alanine aminotransferase. **c** Total bilirubin**. d** INR, international normalized ratio. **e** Albumin. **f** ALP, alkaline phosphatase

**Fig. 2:**
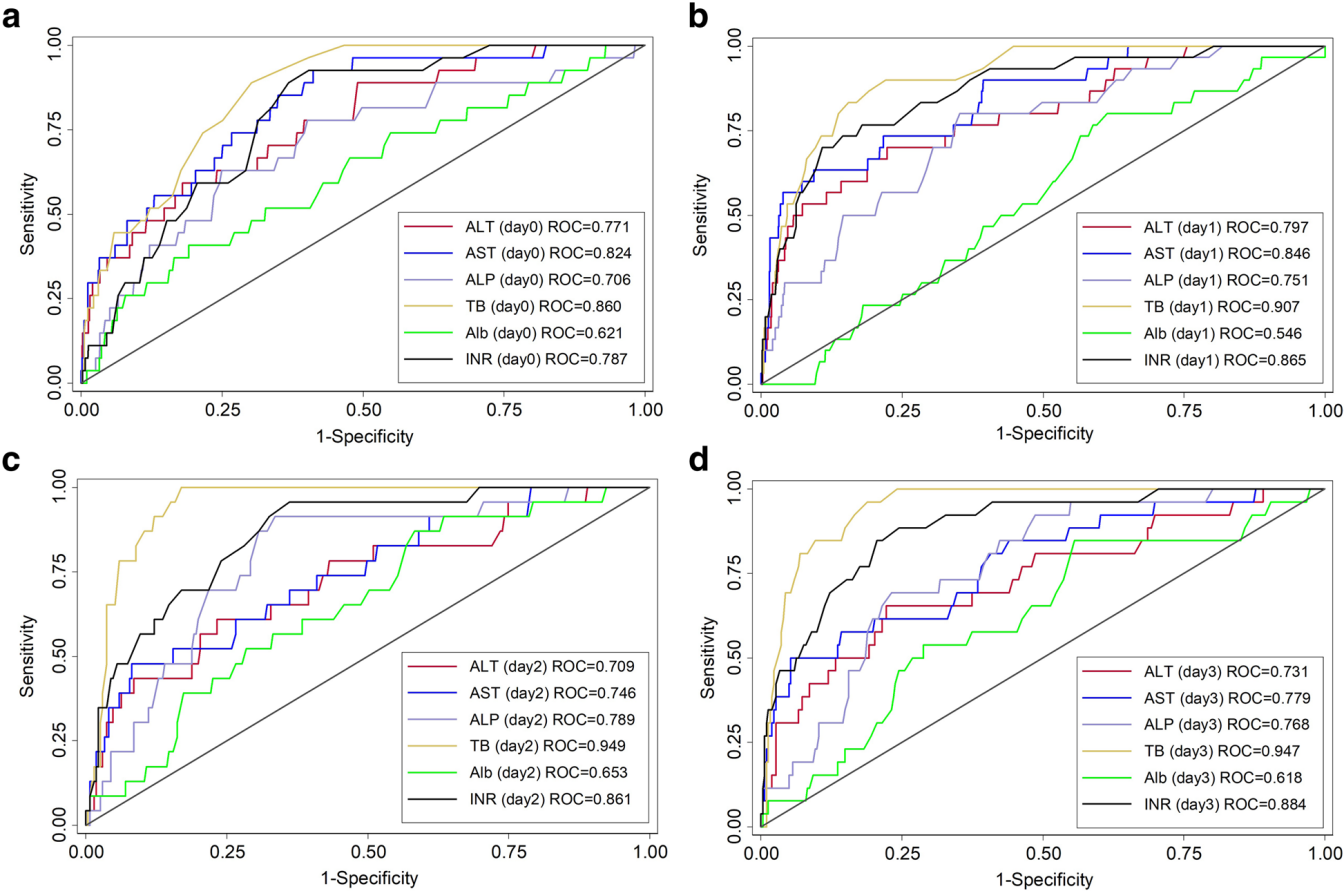
Analysis of predictors among serum transaminase levels by postoperative day. POD, postoperative day; ALT, alanine aminotransferase; AST, aspartate aminotransferase; TB, total bilirubin; INR, international normalized ratio; ALP, alkaline phosphatase; Alb, albumin. **a** POD 0. **b P**OD 1. **c** POD 2. **d** POD 3

The kinetics of postoperative serum transaminase levels, TB, INR, Alb, and ALP in the PHLF group and non-PHLF group are shown in Fig. 1. The peak serum transaminase levels were observed on POD1. The analysis of early postoperative serum AST and ALT cut-off points for prediction of PHLF are shown in Fig. 2. The area under the ROC curve of POD1 serum AST was 0.845 when a cut-off value of 260 U/L was used. The area under the ROC curve for POD1 serum ALT was 0.797 when a cut-off value 270 U/L was used. Postoperative serum AST level had higher accuracy for predicting PHLF than postoperative serum ALT.

### Analysis of risk factors associated with PHLF

The results of the univariate and multivariate analyses of potential early postoperative biochemical risk factors of PHLF are shown in Table 3. Univariate analyses identified the following risk factors for PHLF: female gender (OR 0.4, 95% CI 0.21–0.95; *p* = 0.037), DLP (OR 0.2, 95% CI, 0.05–0.96; *p* = 0.044), smoking (OR 2.7, 95% CI, 1.28–5.72; *p* = 0.009), ICG-R15 (OR 1.0, 95% CI, 1.00–1.06; *p* = 0.031), major hepatectomy (OR 12.5, 95% CI, 5.05–30.83; *p* < 0.001), operative time (OR 1.6, 95% CI, 1.37–1.79; *p* < 0.001), blood loss (OR 1.0, 95% CI, 1.02–1.05; *p* < 0.001), clamp time (OR 1.0, 95% CI, 1.00–1.02; *p* = 0.014), POD1 ALT > 270 U/L (OR 6.5, 95% CI, 2.78–15.38; *p* < 0.001), POD1 AST > 260 U/L (OR 14.0, 95% CI 4.20–46.49; *p* < 0.001), POD1 TB (OR 1.06, 95% CI, 1.01–1.12; *p* = 0.019), POD1 ALP (OR 3.0, 95% CI 1.93–4.78; *p* < 0.001), and POD1 INR (OR 2.6, 95% CI, 2.04–3.42; *p* < 0.001). Multivariate analysis identified the following independent factors associated with PHLF: ICG R15 (OR 1.1, 95% CI, 1.03–1.15, *p* = 0.002), major hepatectomy (OR 6.0, 95% CI, 1.70–20.96; *p* = 0.005), blood loss (OR 1.0, 95% CI 1.00–1.04; *p* = 0.049), POD1 INR (OR 2.0, 95% CI, 1.36–3.00; *p* < 0.001), and POD1 AST > 260 U/L (OR 5.3, 95% CI 1.37–20.83; *p* = 0.016).

**Table 3:** Univariate and multivariate predictors of hepatic failure

Variable	Univariate		Multivariate	
	OR (95% CI)	***p*** value	OR (95% CI)	***p*** value
Age (year)	0.999(0.97–1.03)	0.998		
Gender				
Male	1		1	
Female	0.443(0.21–0.95)	0.037	0.572(0.17–1.94)	0.371
Comorbidity				
DM	0.868(0.35–2.15)	0.760		
HT	0.468(0.21–1.06)	0.069		
DLP	0.227(0.05–0.96)	0.044	0.634(0.13–3.16)	0.579
HBV	1.139(0.50–2.58)	0.756		
HCV	0.754(0.18–3.22)	0.703		
Smoking, *n* = 881				
No	1		1	
Yes	2.714(1.28–5.72)	0.009	1.822(0.57–5.80)	0.310
Preoperative neoadjuvant				
No	1			
Yes	1.564(0.66–3.70)	0.309		
Pre-op diagnosis				
Benign	1			
Malignant	1.976(0.90–4.34)	0.090		
ICG R15, *n* = 537	1.032(1.00–1.06)	0.031	1.091(1.03–1.15)	0.002
Type operation				
Minor	1		1	
Major	12.481(5.05–30.83)	0.000	5.964(1.70–20.96)	0.005
Operative time (h), *n* = 877	1.568(1.37–1.79)	0.000		
Blood loss (ml), *n* = 877	1.032(1.02–1.05)	0.000	1.019(1.00–1.04)	0.049
Clamp time (min), *n* = 620	1.013(1.00–1.02)	0.014		
Pre-op creatinine (mg/dL), *n* = 796	0.691(0.21–2.32)	0.550		
ALBI score, *n* = 819				
ALBI grade I (≤− 2.60)	1			
ALBI grade II (> − 2.60 to ≤ − 1.39)	0.248(0.03–2.07)	0.198		
ALBI grade 3 (> − 1.39)	0.747(0.09–6.21)	0.788		
Pre-op platelet, *n* = 830	1.078(0.72–1.61)	0.711		
POD1 liver function data				
TB (mg/dL) day 1, *n* = 797	1.062(1.01–1.12)	0.019		
ALP (U/L) day 1, *n* = 797	3.036(1.93–4.78)	0.000		
Alb (g/L) day 1, *n* = 825	0.995(0.91–1.09)	0.916		
INR day 1, *n* = 768	2.645(2.04–3.42)	0.000	2.025(1.36–3.00)	0.000
POD1 ALT(U/L), *n* = 784				
ALT ≤ 270	1			
ALT > 270	6.539(2.78–15.38)	0.000		
POD1 AST (U/L), *n* = 796				
AST ≤ 260	1		1	
AST > 260	13.980(4.20–46.49)	0.000	5.346(1.37–20.83)	0.016

*DM* diabetes mellitus, *HT* hypertension, *DLP* dyslipidemia, *HBV* hepatitis B virus, *HCV* hepatitis C virus, *ALBI* albumin-bilirubin, *ICG R15* indocyanine green retention test at 15 min, *TB* total bilirubin, *ALT* alanine aminotransferase, *AST* aspartate aminotransferase, *Alb* albumin, *INR* international normalized ratio

## Discussion

Inadequate functional liver remnant after hepatectomy is the underlying pathophysiology for PHLF (Qadan et al., 2016). Orthotic liver transplantation is considered the best treatment for PHLF, but the shortage of liver donors and the strict inclusion criteria for transplantation are major limitations of this treatment (Søreide & Deshpande, 2021). Therefore, supportive treatment remains the standard of care for PHLF (Søreide & Deshpande, 2021; Qadan et al., 2016). The main components of supportive treatment are early detection and initiation of general care for critically ill patients with focus on organ support, sepsis control, and optimal environment provision for liver generation (Søreide & Deshpande, 2021). Rigorous preoperative assessment and preoperative optimization of patients undergoing hepatectomy are the keys for avoiding PHLF. Preoperative assessment can be carried out using the following parameters: CT volumetric analysis, Child-Pugh classification, evidence of significant portal hypertension, and ICGR15 (Qadan et al., 2016; Walcott-Sapp & Billingsley, 2018). However, PHLF can still occur despite preoperative preparations because of progressively aggressive treatment approaches such as extended hepatectomy, surgery in elderly patients, and hepatectomy following hepatotoxic neoadjuvant chemotherapy (Qadan et al., 2016). The reported incidence of PHLF in current literatures is 1.2–32% (Søreide & Deshpande, 2021; Qadan et al., 2016). From our study, the incidence of PHLF is 3.1%, which is comparable to previous studies.

There are many previous reports on preoperative factors which affects PHLF (Dasari et al., 2019; Chin et al., 2020; Shen et al., 2019; Ye et al., 2020). However, hepatectomy outcomes are also influenced by intraoperative events (Bagante et al., 2019; Grat et al., 2013). Thus, early postoperative parameters would be more accurate than preoperative factors alone for predicting of PHLF (Grat et al., 2013). In the present study, we analyzed preoperative, intraoperative, and postoperative factors. The results showed that a preoperative factor (ICG R15), two intraoperative factors (extent of hepatectomy, blood loss), and some early postoperative parameters (AST, and INR on POD1) were independently associate with PHLF.

According to the ISGLS consensus and the ’50–50’ criteria, PHLF can only be diagnosed on POD5 because the diagnosis is based on biochemical laboratory data taken on POD5 or later (Søreide & Deshpande, 2021). However, waiting until POD5 to make a diagnosis may delay management of patients with PHLF. Therefore, the ability to promptly predict PHLF and deliver early management is crucial to improve the short-term outcomes following hepatectomy (Grat et al., 2013). Regarding serum transaminase, the present study revealed that the serum transaminase levels peaked on POD1. Consistently, Higaki et al. (Higaki et al., 2018) examined the association between ischemic parenchymal volume of the liver after hepatectomy and serum transaminase elevation, and found that serum transaminase level in their cohort also peaked on POD1. In addition, the present ROC analysis of serum transaminase level from POD0 to POD3 revealed that the transaminase level on POD1 has the highest yield for prediction of PHLF. These findings are consistent with those in a study conducted by Grat et al. (Grat et al., 2013) who analyzed POD1 serum biochemical parameters in patients after major liver resection for colorectal metastases. They found that an AST cut-off point of 798 U/L on POD1 can stratify patients into low-risk and high-risk groups for 90-day mortality. Olthof et al. (Yoshino et al., 2021) also retrospectively studied patients who underwent liver resection and found that peak AST level, which normally occurs within 24 h after hepatectomy, of > 828 U/L associated with increased postoperative morbidity and mortality. The higher cut-off point found by Grat et al. compared with the value of 250 U/L in the present study can possibly be explained by the difference in primary outcomes. Specifically, the primary outcome in present study is PHLF while the primary outcomes in the other study were overall mortality and morbidity.

In contrast to the present findings, Bhogal et al. (Bhogal et al., 2016) reported that serum ALT on POD1 was not predictive of post-hepatectomy morbidity and mortality. However, they did not investigate the ability of elevated AST as a predictor. In the present study, AST was associated with PHLF, but not ALT. Another contradictive work is a study by Boleslawski et al. (Boleslawski et al., 2014), who found that post-hepatectomy serum AST and ALT were not independently associate with morbidity. However, their definition of postoperative morbidity was inclusive of all manner of complications, including pulmonary complications, hemorrhage, wound infections, with only 3% of the reported complications were PHLF.

Preoperative AST was found to be better than preoperative ALT for predicting outcomes following hepatectomy in previous reports (Ye et al., 2020; Liu et al., 2020; Saadat et al., 2021; Shi et al., 2021). Liu et al. (Liu et al., 2020) and Saadat et al. (Saadat et al., 2021) conducted large-population studies on the preoperative factors of PHLF, and found that preoperative AST > 40 U/L was associated with PHLF. Postoperative AST had also reported to be more strongly associated with postoperative outcomes than postoperative ALT (Olthof et al., 2016; Grat et al., 2013). Olthof et al. (Olthof et al., 2016) reported that peak postoperative AST, but not ALT, was associated with the overall postoperative morbidity and mortality. Consistently, Grat et al. (Grat et al., 2013) reported that postoperative AST, INR, and bilirubin were associated with 90-day mortality. Meanwhile, AST alone was not significantly associated with overall hepatic complications, including PHLF, delayed recovery of liver function, bile leakage, and subphrenic abscess. Yu et al. (Yu et al., 2018) reported that among PHLF patients, sustained ALT elevation beyond POD1 was associated with increased mortality. However, the observation was only made within a PHLF cohort with no comparison against a non-PHLF cohort. To the best of our knowledge, there are no previous reports on the association of peak postoperative serum transaminase levels with PHLF as the primary outcome.

From the present result with PHLF is the primary outcome, only serum AST > 250 U/L on POD1 had significant association with PHLF, while ALT did not. An explanation for the association of AST with PHLF may be hepatocellular injury, which can be caused by multiple factors (Søreide & Deshpande, 2021; Qadan et al., 2016; Murtha-Lemekhova et al., 2021). Aminotransferases (also known as transferases) are enzymes involved in the transfer of amino groups from aspartates to ketoglutaric acid and are markers of hepatocellular injury (Robles-Diaz et al., 2015). Elevated levels of AST and/or ALT, ALP, and bilirubin can suggest the occurrence of hepatocellular injury and are associated with increased liver-related mortality in the general population, as well as in post-hepatectomy patients (Kwo et al., 2017). In addition, plasma transaminase levels are measured after liver surgery as markers of hepatocellular injury and have been used as endpoints in numerous previous clinical trials (Murtha-Lemekhova et al., 2021; Beck-Schimmer et al., 2012; Nguyen et al., 2019). However, the mechanism of post-hepatectomy hepatocellular injury dependent on multi-factors. Some of these factors were (1) preexisting conditions (such as hepatic steatosis, fibrosis, or cirrhosis) that can reduce liver regeneration capacity, (2) vascular inflow occlusion during the operation, and (3) ongoing injury due to hyperperfusion in a proinflammatory environment (Murtha-Lemekhova et al., 2021). Thus, further large-population prospective studies on the relationship between serum AST or ALT and PHLF should be conducted to confirm the findings of the present study.

The present results also showed that INR on POD1 was independently associated with PHLF. INR was shown to temporarily increase after hepatectomy in some patients, with a peak on POD1 or 2 (Balzan et al., 2005). Usually, INR gradually normalize on POD3 or 4 (Martin 2nd et al., 2003). However, those with PHLF had sustained elevation after POD5, leading to the currently accepted criteria for diagnosis of PHLF (Rahbari et al., 2011). Although non-PHLF patients can have elevated INR level on POD1, high INR on POD1 was demonstrated by Roberts et al. (Roberts et al., 2013) to signify increasing severity of PHLF. These findings are similar to the present findings and suggest that INR on POD1 can be an early warning for physician that PHLF may be underway.

There are a few limitations to the present study. First, because of its retrospective nature, some selection bias may have been present. Second, the characteristics of patients undergoing hepatectomy can be heterogenous, and there was a lack of data on the degree of background liver disease, postoperative serum glucose, and serum lactate level in the study. Third, the small population of the study, which there were relatively small number of PHLF patient which would affecting the power of the analysis. In addition, there were only four patients who died from PHLF, and thus multiple logistic regression analyses could not be performed to evaluate the use of the identified parameters for prediction of mortality.

## Conclusion

Postoperative serum AST, TB, and INR level on POD1 were found to be independently associated with PHF as well as ICG R15, and major hepatectomy. Such factors can be determined as early as POD1. Thus, the prompt warning can help alert physicians that the patient is at risk so that active management and vigilant monitoring such as more frequent blood tests, more invasive or advanced circulatory monitoring (e.g., central venous catheterization, pulmonary artery catheterization, stroke volume variation monitoring) or the transfer to an ICU can be started earlier.

## Data Availability

The datasets used and/or analyzed during the current study are available from the corresponding author on reasonable request.

## Abbreviations

PHLF: Post-hepatectomy liver failure
ISGLS: International Consensus of the Liver Surgery Group
CT: Computed tomography
MRI: Magnetic resonance imaging
CBC: Complete blood count
LFT: Liver function tests
ALBI: Albumin-bilirubin
ICG-R15: Indocyanine green retention test at 15 min
POD: Postoperative day
DLP: Dyslipidemia
HBV: Hepatitis B virus
ALT: Alanine aminotransferase
AST: Aspartate aminotransferase
ALP: Alkaline phosphatase
Alb: Albumin
PO_4_: Phosphate
OR: Odds ratio
CI: Confidence interval
ROC: Receiver operating characteristic
